# Task-order control in dual-tasks: Only marginal interactions between conflict at lower levels and higher processes of task organization

**DOI:** 10.3758/s13414-024-02876-9

**Published:** 2024-05-08

**Authors:** Valentin Koob, David Dignath, Markus Janczyk

**Affiliations:** 1 Research Methods and Cognitive Psychology, Department of Psychology, University Bremen, Hochschulring 18, D-28359 Bremen, Germany; 2 Eberhard Karls University of Tübingen, Tübingen, Germany

**Keywords:** Dual-tasking, Backward crosstalk, Sequential modulation, Task-order control

## Abstract

When simultaneously performing two tasks that share response properties, interference can occur. Besides general performance decrements, performance in the first task is worse when the second task requires a spatially incompatible response, known as the backward crosstalk effect (BCE). The size of this BCE, similar to congruency effects in conflict tasks, is subject to a sequential modulation, with a smaller BCE after incompatible compared to compatible trials. In the present study, we focus on a potential bidirectional interaction between crosstalk (and its resolution) at a lower level of task performance and higher-order processes of task organization. Two questions were of particular interest: First, do participants switch task order more frequently after a conflict-prone incompatible trial than after a compatible trial? Second, does changing task order influence the efficiency of conflict resolution, as indexed by the size of the sequential modulation of the BCE. Across four experiments, we only found marginal evidence for an influence of lower-level conflict on higher-order processes of task organization, with only one experiment revealing a tendency to repeat task order following conflict. Our results thus suggest practical independence between conflict and task-order control. When separating processes of task selection and task performance, the sequential modulation was generally diminished, suggesting that conflict resolution in dual-tasks can be disrupted by a deliberate decision about task order, or, alternatively, by a longer inter-trial interval. Finally, the study found a strong bias towards repeating the same task order across trials, suggesting that task-order sets not only impact task performance but also guide task selection.

People often plan and perform multiple tasks to reach their goals (Cooper & Shallice, 2000; Humphreys & Forde, 1998; Keele & Cohen, 1990). Specifically, people must decide how and which tasks to perform (Brüning et al., 2020; Damos et al., 1983; Kahneman, 1973), and then have to perform the selected tasks either concurrently or sequentially. It is not well understood whether and how agents consider information about task performance (i.e., how well they did something) for their choices (i.e., what to do next). The present study investigates this question in the context of dual-tasking. In particular, we ask how decision-making in dual-tasking modulates task performance and how crosstalk during task performance guides decision-making.

## Hierarchical control in dual-tasking

A useful way to study task performance in dual-tasking are psychological refractory period (PRP) experiments, where participants perform two tasks (i.e., Task 1 and Task 2) in rapid succession. The observation that response times (RTs) in Task 2 usually increase the closer both tasks are presented in time, while performance in Task 1 often remains unaffected, has been termed the PRP effect (Telford, 1931; Welford, 1952). The PRP effect can be explained with the *response selection bottleneck* (RSB) model (Pashler, 1984, 1994). This model assumes that peripheral processes, such as stimulus perception and motor execution, run in parallel to processes of other tasks, while central processes of response selection can only be utilized by one task at any time. Thus, these stages have to proceed sequentially and when the central bottleneck is already occupied, response selection in Task 2 is postponed (see Fischer and Janczyk, 2022, for a recent overview).

As subtasks compete for central processing in any dual-task setting, the scheduling of task order becomes necessary. Although the original RSB model suggests task order scheduling in terms of central arrival time (i.e., a ’first-come-first-serve’ principle), several studies provide evidence for dedicated mechanisms for this purpose (De Jong, 1995; Logan and Gordon, 2001; Luria and Meiran 2003, 2006; Sigman and Dehaene, 2006; see also Schubert, 2008). For example, in Experiment 1 of De Jong (1995), participants were presented with a cue indicating the temporal order of tasks in an upcoming trial. In some trials, however, the actual order of stimuli was different, causing an increase in reversals of response order. Based on these results, De Jong concluded that task order in a dual-task setting can be planned ahead. To date, multiple behavioral (Kübler et al., 2018; Luria & Meiran, 2003, 2006) and neurophysiological (Kübler et al., 2019; Stelzel et al., 2008; Szameitat et al., 2006) studies support De Jong ’s conclusion and provide evidence for a separate representation guiding the order of subtasks. This representation is called the *task-order set*, and once active in working memory (Kübler et al., 2022b), it persists across successive trials, requiring modification if task order switches. As a result, performance is better in trials with a repeating relative to a changing task order, and this difference is referred to as task-order switch costs.

Task-order switch costs also indicate that higher-order information, such as the task-order set, can influence performance. To account for this hierarchical relationship (see also Botvinick, 2008; Cooper and Shallice, 2000), theoretical models of multitasking (Kieras & Meyer, 1997; Rubinstein, 2001; Sigman & Dehaene, 2006), such as the executive control theory of visual attention (ECTVA; Logan and Gordon, 2001), assume that dual-task performance results from the interplay of two distinct levels that are part of the working memory: a task and a parameter level. The task level consists of instructions and stimulus-response (S-R) rules and is associated with task selection. The subordinate parameter level uses input from the task level to set control parameters for the specific S-R rules and is associated with task performance. Such a hierarchical view is further corroborated by behavioral (e.g., Kleinsorge and Heuer, 1999; Schneider and Logan, 2006; Weaver and Arrington, 2013) and neurophysiological evidence (e.g., Braverman et al., 2014; Steinhauser and Steinhauser, 2018; see also Badre, 2008; Koechlin and Summerfield, 2007). The present research adopts such a hierarchical view and investigates whether lower-level processes related to between-task response interference during subtask performance interact with higher-level processes active during task order scheduling. Therefore, we will first introduce the concept of response compatibility in dual-task performance, before we turn to research on task selection in multitasking, and finally explain the rationale of the present study.

## Response compatibility in dual-tasking

Between-task response interference can be observed in many dual-task setups. For example, the participants of Hommel (1998, Exp. 1) were presented with a colored letter and asked to respond to the letter’s color with a left or right hand response in Task 1 (R1) and to the letter’s identity by uttering ’left’ or ’right’ in Task 2 (R2). A trial was considered R1-R2 compatible if the required responses R1 and R2 matched in their spatial feature, and it was considered R1-R2 incompatible if both mismatched. The important result was that R1s were faster and less error-prone in compatible trials relative to incompatible trials. This is remarkable, as it suggests that Task 2 response information is activated, at least to some degree, while Task 1 processing is still ongoing. The present study focuses on this effect, which we refer to as the *R1-R2 backward compatibility (or crosstalk) effect (BCE)*, that has been replicated multiple times (Durst & Janczyk, 2019; Ellenbogen & Meiran, 2011; Fischer et al., 2007; Janczyk, 2016; Janczyk, Renas et al., 2018; Koob et al., 2020; Lien et al., 2006; Logan & Schulkind, 2000; Miller & Alderton, 2006; Thomson et al., 2010, 2015).

According to Janczyk, Renas et al., (2018), the R1-R2 BCE has its locus in the response selection stage of Task 1 (see also Miller, 2017; Thomson et al., 2015). In particular, it is hypothesized that the stimulus of Task 2 activates its associated response after perception. Thus, given sufficient temporal proximity between the two stimuli, activated Task 2 response information can then influence Task 1 response selection, thereby shortening or prolonging Task 1 response selection on compatible or incompatible trials, respectively (Koob et al., 2024; see also Koob, Ulrich et al., 2023, for a computational approach).

Another important observation for the present study is the modulation of the BCE by the compatibility of the previous trial. More specifically, the size of the BCE is reduced after conflict-prone incompatible trials compared to compatible trials (Durst and Janczyk, 2019; Janczyk, 2016; Janczyk et al., 2017; Janczyk, Mittelstädt et al., 2018; Renas et al., 2018; Scherbaum et al., 2015; Schonard et al., 2020; see Schuch et al., 2019, for a review and integrated view). This effect has been explained by an attentional shift from Task 2 to Task 1 (see also Lehle and Hübner, 2009; Miller and Tang, 2021) after experiencing an incompatible trial, leading to less pre-activated Task 2 response information being present. Yet, how exactly incompatible trials trigger this adaptation is not well understood. For similar sequential modulations of congruency effects found with single tasks (Gratton et al., 1992), it has been suggested that conflict between responses triggers a learning signal that strengthens task-relevant over task-irrelevant processes (Botvinick et al., 2001; Verguts and Notebaert, 2008; see also Koob, Mackenzie et al., 2023). However, theoretical views regarding the nature of this learning signal from conflict differ (e.g., Botvinick et al., 2001; Dignath et al., 2020; Gratton et al., 1992; Schmidt, 2013; see also Schonard et al., 2020, for a discussion in the context of dual-tasks). Yet, despite these differences, theoretical views converge on the assumption that the sequential modulation of conflict effects provides a window into adaptive control processes (Egner, 2014). Following this perspective, the present research uses the relative size of the sequential BCE as an index of adaptive control in dual-tasking (see also Fischer et al., 2014, for a similar logic).

## Task choice in voluntary task switching and dual-tasking

In many real-life situations, people not only have to execute relevant tasks correctly, but they also have to schedule the order of tasks accordingly. Arrington and Logan (2005) introduced the voluntary task switching paradigm, a protocol which allows to assess both task selection and task performance simultaneously. In such experiments, participants choose randomly among two tasks on each trial and then perform the selected task (Arrington & Logan, 2004, 2005). These and subsequent experiments showed that participants repeat the same task in the majority of trials (for a summary, see Arrington et al., 2014). To account for this repetition bias, Arrington et al. suggested that participants mix two different strategies when choosing tasks. A representativeness heuristic reflects participants’ conception of randomness, while an availability heuristic suggests selecting the most active task, which is often the most recently performed task.

Interestingly, the activation of a task is not only modulated by bottom-up factors like task availability, but also by top-down factors, like cognitive control. For instance, task switching studies reported evidence that conflict in incongruent trials biases subsequent task selection (e.g., Dignath et al., 2015; Naefgen et al., 2022; Orr et al., 2012). Orr et al. (2012) found a stronger bias to repeat a task after conflict when using two tasks that share the same S-R sets (see also Dignath et al., 2015, Exp. 2). Using two tasks with different S-R sets, however, Dignath et al. (2015) observed a tendency to switch tasks after conflict, potentially as a strategy to escape the situation creating this conflict. Similarly, Naefgen et al. (2022) demonstrated that participants avoid making an incompatible response in dual-task settings.

## The present study

The present study investigates how task selection modulates task performance and how crosstalk during task performance influences task selection in a dual-task setting. It was motivated by a recent framework of cognitive control in multitasking (Schuch et al., 2019), proposing that conflict-control loops reflect a general principle of the cognitive system, and that conflict should modulate behavior at multiple levels. In all of the following experiments, participants were instructed to choose the order of two tasks with overlapping response properties, and then perform both tasks in quick succession. The following hypotheses were considered.

First, we expect that task selection will affect task performance. More specifically, we predict that repeating versus switching task order modulates the efficiency of conflict resolution, as indicated by a sequential modulation of the BCE. Based on previous research on task-order control in dual-tasking (De Jong, 1995; Luria & Meiran, 2003, 2006) and cognitive control in single-tasking (Braem et al., 2014; Dignath et al., 2019; Kiesel, 2006; Kreutzfeldt et al., 2016; Spapé & Hommel, 2008), we assume that task order provides a distinct representation (i.e., a ’context’) that makes it easier to exert control if it is repeated compared to switched. More precisely, it is assumed that different concrete and abstract properties present during a trial, such as stimuli, responses, control states, or detected conflict, are associated with each other and stored in a so-called episodic file (Egner, 2014). If the context repeats, the previously formed episodic file can be retrieved, and corresponding cognitive control states (re-)instantiated. Thus, the sequential modulation of the BCE could be larger for task-order repetitions compared with task-order switches.

Second, following the idea that conflict resolution might act on different levels, we expect conflict during task performance to affect task selection. However, predictions are not straightforward. (1) Recent versions of conflict monitoring theory predict that conflict should strengthen all currently active task representations (Verguts & Notebaert, 2008). According to this perspective, conflict might also strengthen the task-order set, increasing its relative availability for the upcoming trial. If so, we would expect a conflict in the previous trial to bias the decision towards task-order repetitions. (2) Conflict could also trigger a motivation to avoid the same situation creating this conflict in the next trial (Dignath et al., 2015). Thus, participants might explore options to change the task order after a conflict-prone incompatible trial. In this case, we expect more task-order switches after an incompatible trial. (3) A last possibility is, of course, that conflict between tasks may not influence task order at all. Recent research suggests that the task-order set comprises only abstract information about the order of subtasks and not the specific S-R translations Kübler et al. (2022b), making it unlikely for conflict signals at the lower level of task performance to affect higher-level processes of task selection.

Four experiments are reported which systematically varied the overlap between task selection and task performance (Experiments 1a & Exp. 2a: simultaneous registration of task selection and performance; Experiments 1b & Exp. 2b: independent registration of task selection and performance) and the degree of stimulus separation during task performance (Experiment 1a & 1b: integral stimulus presentation; Experiments 2a & Exp. 2b: separate stimulus presentation).^1^ In all experiments, participants were asked to complete two (sub)tasks on each trial, but were free to choose the order of these tasks.

## Experiment 1a

### Method

#### Participants

Twenty-four students (18 female) from the University of Tübingen, aged 18 to 30 years (M = 21.96 years, SD = 3.75), participated for monetary compensation (8€) or course credit. All participants provided written informed consent prior to data collection and had normal or corrected-to-normal vision. Four participants were excluded because of a high tendency of repeating the same task order ($> 90 \%$; see Design and Analysis for further information).

#### Apparatus and stimuli

A standard PC was used for stimulus presentation and response collection. Stimuli and instructions were presented on a 17-in. CRT monitor. The letters ‘S’ or ‘X’ presented in red or green served as stimuli. Responses to the letter’s identity were manual key presses with the response keys placed to the left and right of the participant on the table. Responses to the letter’s color were pedal presses on one of two foot-pedals, one underneath the left and one underneath the right foot of the participant on the floor.

#### Task and procedure

Every trial started with a white fixation cross (250 ms), followed by a blank screen (250 ms). Subsequently, the colored letter was presented for a maximum of 4000 ms or until both responses were registered. The next trial started after an inter-trial interval (ITI) of 1000 ms. In case of an error, specific error feedback was provided for 1000 ms before the ITI.

Participants first performed a short familiarization block of 20 randomly drawn trials, which was followed by ten experimental blocks of 60 trials each, resulting from 15 repetitions of all combinations of 2 S1 × 2 S2. All trials were presented in a random order. Participants received written instructions that emphasized speed and accuracy. The task order was unconstrained. Thus, in each trial, participants were free to perform either the manual task or the pedal task first. Participants were instructed to choose both possible task orders about equally often. The S-R mappings of both tasks were counterbalanced across participants.

#### Design and analysis

Trials *N* in which subtasks were carried out in the same order as in the previous trial $N-1$ were considered task-order repetitions, whereas trials in which the task order changed were considered task-order switches. Trials in which both responses were to-be-given on the same side (i.e., left index finger and left foot, or right index finger and right foot) were considered (response) compatible, otherwise they were considered (response) incompatible. Data were pre-processed in the following steps: Data from the familiarization block were discarded first. Afterwards, we checked if any participant provided an erroneous trial in more than $20\%$ (which was not the case here), before discarding the first trial of each block. Then, we discarded trials with a general error in trial *N* (e.g., responses faster than 100 ms, time-outs; $1.33\%$) and checked if any participant provided an inter-response interval of less than 50 ms in more than $20\%$ of the trials (which was not the case here). We then proceeded by discarding general errors in trial $N-1$ ($1.33\%$), and current trials or trials following trials with an inter-response interval of less than 50 ms ($10.35\%$). Based on these data, we then excluded four participants who changed task order in less than $10\%$ of the trials. Finally, we excluded trials with a response error in at least one of the tasks in trial $N-1$ for all analyses ($6.84\%$), as is typical when investigating sequential modulations of the BCE (e.g., Janczyk, 2016). Overall, we considered three dependent variables: RTs and percentages error (PEs) of the first performed task, and task-order switch (TOS) rates. For RT analyses, only correct trials *N* were considered (discarding $6.2\%$ of the trials) and trials where RTs deviate more than 2.5 standard deviations (*SD*s) from the individual cell mean were discarded as outliers ($2.56\%$). RTs and PEs of the second performed task were not analyzed.

Mean RTs and PEs of the first performed task were submitted to separate $2 \times 2 \times 2$ analyses of variance (ANOVAs) with the factors compatibility in trial *N* (compatible vs. incompatible), compatibility in trial $N-1$ (compatible vs. incompatible), and task-order switch (switch vs. repetition) as repeated measures. TOS rates were analyzed by contrasting them after compatible and incompatible trials $N-1$ using a standard paired *t* test. The classical (frequentist) *t* test for TOS rates was complemented by a Bayesian *t* test using the function *ttestBF* from the R package *BayesFactor* (Morey and Rouder, 2022, v. 0.9.12-4.4). This was done because we had no hypothesis about the influence of compatibility in trial $N-1$ on TOS rates, and the corresponding Bayes factors might help to update our believes about the presence or absence of an effect. For each statistical test, we ensured that each participant provided at least 6 trials for each experimental cell.^2^

### Results

**Fig. 1:**
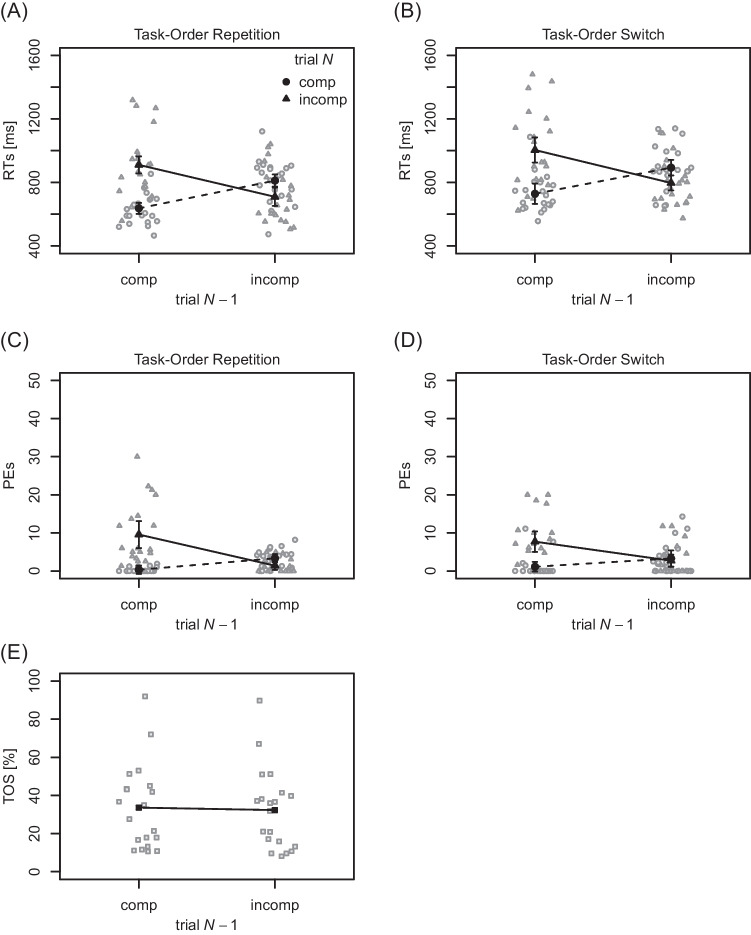
Mean response times, percentages error, and task-order switch rates for Experiment 1a. *Note.* (**A**) and (**B**) Results for response times (RTs) to the first performed task as a function of compatibility in trial *N* and compatibility in trial $N-1$, separately for task-order repetitions and switches, respectively. (**C**) and (**D**) Analogous results for percentages error (PEs). (**E**) Task-order switch (TOS) rates as a function of trial $N - 1$ compatibility. In panels (**A**) to (**D**), *gray dots* and *triangles* reflect individual mean RTs/PEs for compatible and incompatible trials *N*, respectively. In panel (**E**), *gray squares* indicate individual mean TOS rates. Across all panels, *black dots*, *triangles*, and *squares* indicate mean values, aggregated across the corresponding individual data points. *Error bars* around the mean values indicate $95\%$ confidence intervals after removing inter-subject variability (Morey, 2008; too small to see in panel **E**); comp = compatible, incomp = incompatible

**RTs** Mean and individual RTs of the first performed task are visualized in Fig. 1A and B. RTs were shorter in compatible (768 ms) relative to incompatible (855 ms) trials *N*, $F(1, 19) = 9.12$, $p = .007$, $\eta _p^2 = .32$, thus a BCE was present. The main effect of compatibility in trial $N-1$ missed the conventional level of statistical significance, $F(1, 19) = 3.27$, $p = .086$, $\eta _p^2 = .15$, although descriptively RTs were slightly longer following compatible (820 ms) relative to incompatible (803 ms) trials. The main effect of task-order switch was significant, $F(1, 19) = 12.28$, $p = .002$, $\eta _p^2 = .39$, with RTs being shorter in task-order repetitions (767 ms) relative to switches (856 ms). The interaction of compatibility in trial *N* and in trial $N-1$ was significant, $F(1, 19) = 86.99$, $p < .001$, $\eta _p^2 = .82$, indicating a sequential modulation. In particular, the BCE was clearly present following compatible trials $N-1$ (275 ms), and it was inverted following incompatible trials $N-1$ ($-99$ ms). None of the remaining interactions reached statistical significance, $F\text {s} \le 0.25, p\text {s} \ge .626$.

**PEs** Mean and individual PEs of the first performed task are visualized in Fig. 1C and D. They were lower in compatible ($2.02 \%$) relative to incompatible ($5.34 \%$) trials *N*, $F(1, 19) = 17.08$, $p = .001$, $\eta _p^2 = .47$, thus a BCE was present. The main effect of compatibility in trial $N-1$ was statistically significant, $F(1, 19) = 8.02$, $p = .011$, $\eta _p^2 = .30$, with PEs being higher after compatible ($4.68\%$) relative to incompatible trials ($2.68\%$). The main effect of task-order switch was not statistically significant, $F(1, 19) < 0.01$, $p = .951$, $\eta _p^2 < .01$. The interaction of compatibility in trial *N* and in trial $N-1$ was significant, $F(1, 19) = 63.32$, $p < .001$, $\eta _p^2 = .77$, indicating a sequential modulation. The BCE was clearly present following compatible trials $N-1$ (7.91 percentage points), and it was inverted following incompatible trials $N-1$ ($-1.27$ percentage points). All remaining interactions did not reach statistical significance, $F\text {s} \le 2.40, p\text {s} \ge .138$.

**TOS rates** Mean and individual TOS rates are visualized in Fig. 1E. The overall TOS rate of $32.95\%$ was significantly different from $50 \%$, $t(19) = -3.51$, $p = .002$, $d = -0.78$. TOS rates were slightly smaller for incompatible ($32.3\%$) relative to compatible ($33.6\%$) trials $N-1$. Yet, the corresponding *t* test did not reach statistical significance, $t(19) = 1.88$, $p = .075$, $d = .42$, and the complementary Bayes factor remained indecisive, $BF_{01} = 0.99$ $(\pm 0.02\%)$.^3^ Since Bayes factors are sensitive to the parametrization of priors, we reran our analysis with wider priors on the standardized effect size, using the pre-specified labels of ’wide’ and ’ultrawide’ for the *rscale* argument in the *BayesFactor* R-Package (instead of the default label ’medium’). As can be expected, Bayes factors tended to be higher with wider prior distributions, although they only reached a value of $BF_{01} \!=\! 1.59$ $(\pm \!<\! 0.01\%)$. Thus, even with wider priors, Bayes factors remained indecisive, suggesting that we do not have enough data to unequivocally support a certain conclusion.

### Discussion

We observed faster and less error-prone responses in compatible trials *N* relative to incompatible trials *N*, that is, a BCE was observed. This BCE was smaller and even reversed after an incompatible relative to a compatible trial $N-1$, reflecting the typical sequential modulation of the BCE (Durst & Janczyk, 2019; Janczyk, 2016; Schonard et al., 2020). Yet, the analyses did not reveal evidence for a reduced sequential modulation after task-order switches relative to repetitions, that is, for an influence of task selection processes on conflict resolution.

Overall, we observed a strong tendency to repeat the same task order across successive trials. Regarding the influence of conflict in trial $N-1$ on processes of task selection, the picture is not very clear. Descriptively, we observed TOS rates to be slightly higher after compatible trials. Yet, this difference was relatively small in its absolute values ($~ -1.3$ percentage points), the corresponding classical *t* test turned out non-significant, and the complementary Bayes factors were indecisive regarding the absence or presence of an effect. Thus, at present, we cannot draw a definite conclusion about a potential influence of (between-task) response conflict at a lower level of task performance on higher-order processes of task selection.

## Experiment 1b

Experiment 1b was similar to Experiment 1a with one change. Participants were now required to indicate their task-order choice prior to each trial (see, e.g., Arrington and Logan, 2005, Exp. 6; Dignath et al., 2015). This additional manipulation was introduced to separate processes of task choice and task performance and to increase the salience of task orders and their switches. In particular, as was mentioned in the Introduction, changing properties of a task (i.e., the ’context’) between two trials can interrupt the retrieval of control states from the previous trial (e.g., Dignath et al., 2019; Spapé and Hommel, 2008), which might lead to a reduced sequential modulation of the BCE after task-order switches (which was not the case in Experiment 1a). Furthermore, since announcing one’s task order choice prior to a trial naturally increases the time between trials, this might allow participants to better adapt their task-order choice in response to a previously experienced conflict.

### Method

#### Participants

Twenty-four students (12 female) from the University of Tübingen, aged 19 to 26 years ($M = 21.7$ years, $SD = 2.3$), participated with the same criteria as for Experiment 1a. Two participants were excluded for committing a response error or a general error (e.g., response too early, too slow) in more than $20\%$ of the trials.

#### Apparatus, stimuli, and tasks

The apparatus, stimuli, and tasks were the same as in Experiment 1a.

#### Procedure

Every trial started with the presentation of a white “?” in the center of the screen. Participants then indicated their task-order choice for the present trial via two simultaneous presses of the manual or pedal keys. In other words, two manual key presses logged the manual task as the first task, whereas two pedal presses logged the pedal task as the first task for the present trial. After the participant released the keys, a blank screen was shown for 1500 ms, before the trial proceeded as in Experiment 1a.

#### Design and analysis

The design, data analyses, and data pre-processing were similar to those of Experiment 1a. We excluded two participants for committing a response error or a general error in more than $20\%$ of the trials.^4^ Trials with a general error in trial *N* were discarded in $5.81 \%$ of the trials, trials following a general error in $5.67\%$, and trials with an inter-response interval of less than 50 ms in trial *N* or trial $N-1$ in $1.28\%$. Finally, we excluded trials following a response error in at least one of the tasks for all analyses ($5.26\%$). For RT analyses, only correct trials *N* were considered (thus discarding $5.44\%$ of the trials) and another $2.92\%$ of the trials were discarded as outliers. Finally, we had to exclude one participant for RT and PE analyses as this participant did not provide at least six trials in all cell combinations of trial *N* compatibility, trial $N-1$ compatibility, and task-order switch.

### Results

**Fig. 2:**
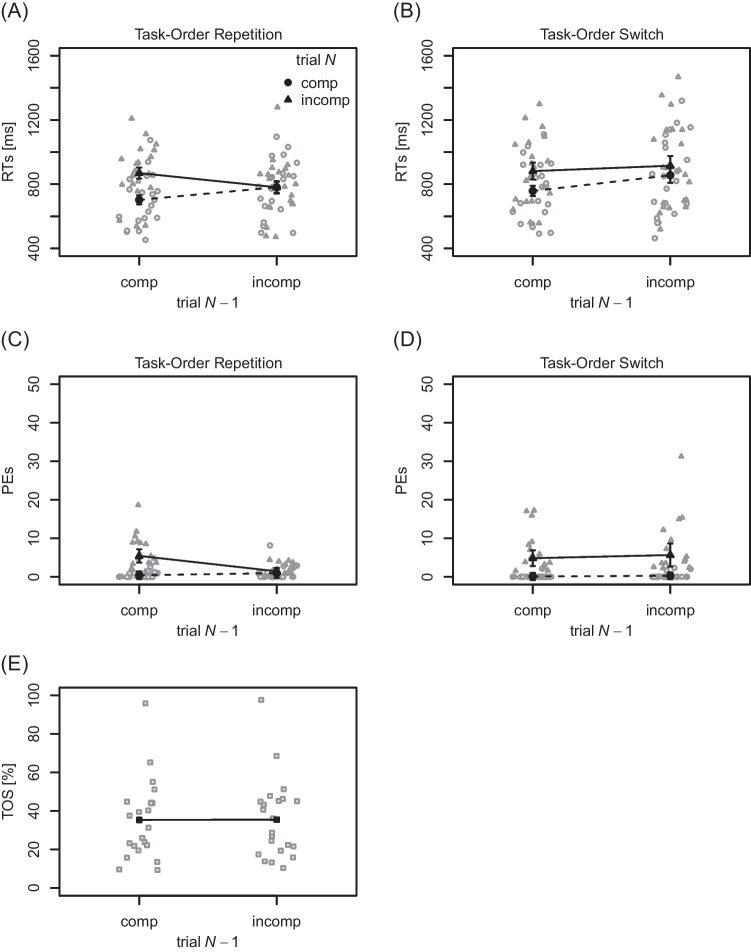
Mean response times, percentages error, and task-order switch rates for Experiment 1b. *Note.* (**A**) and (**B**) Results for response times (RTs) to the first performed task as a function of compatibility in trial *N* and compatibility in trial $N-1$, separately for task-order repetitions and switches, respectively. (**C**) and (**D**) Analogous results for percentages error (PEs). (**E**) Task-order switch (TOS) rates as a function of trial N - 1 compatibility. In panels (**A**) to (**D**), *gray dots* and *triangles* reflect individual mean RTs/PEs for compatible and incompatible trials *N*, respectively. In panel (**E**), *gray squares* indicate individual mean TOS rates. Across all panels, *black dots*, *triangles*, and *squares* indicate mean values, aggregated across the corresponding individual data points. *Error bars* around the mean values indicate $95\%$ confidence intervals after removing inter-subject variability (Morey, 2008; too small to see in panel **E**); comp = compatible, incomp = incompatible

**RTs** Mean and individual RTs to the first performed task are visualized in Figs. 2A and B. RTs were shorter in compatible (774 ms) relative to incompatible (862 ms) trials *N*, $F(1, 20) = 34.41$, $p < .001$, $\eta _p^2 = .63$, thus a BCE was present. The main effect of task-order switch was significant, $F(1, 20) = 9.26$, $p = .006$, $\eta _p^2 = .32$, with RTs being shorter when task order repeated (783 ms) than when it switched (852 ms). The main effect of compatibility in trial $N-1$ was statistically significant, $F(1, 20) = 5.51$, $p = .029$, $\eta _p^2 = .22$, with RTs being longer following incompatible (833 ms) than compatible (803 ms) trials. However, this difference between previously compatible and incompatible trials was primarily present for task-order switches (66 ms) but not for task-order repetitions ($-5$ ms), as indicated by a significant two-way interaction between trial $N-1$ compatibility and task-order switch, $F(1, 20) = 6.61$, $p = .018$, $\eta _p^2 = .25$. The interaction of compatibility in trial *N* and trial $N-1$ was significant, $F(1, 20) = 29.14$, $p < .001$, $\eta _p^2 = .59$, indicating a sequential modulation. In particular, the BCE was larger following compatible trials $N-1$ (145 ms) relative to incompatible trials $N-1$ (30 ms). Importantly, this two-way interaction was further qualified by the factor of task-order switch, $F(1, 20) = 6.20$, $p = .022$, $\eta _p^2 = .24$. Whereas the sequential modulation of the BCE was clearly present for task-order repetitions (167 ms), it was reduced for task-order switches (64 ms). The remaining two-way interaction between trial *N* compatibility and task-order switch was not statistically significant, $F(1, 20) = 0.19$, $p = .671$, $\eta _p^2 = .01$.

**PEs** Mean and individual PEs are visualized in Fig. 2C and D. They were smaller for compatible ($0.45 \%$) relative to incompatible ($4.35 \%$) trials *N*, $F(1, 20) = 23.10$, $p < .001$, $\eta _p^2 = .54$, thus a BCE was present. The main effect of compatibility in trial $N-1$ was not statistically significant, $F(1, 20) = 1.80$, $p = .195$, $\eta _p^2 = .08$, although PEs were slightly higher after compatible ($2.71\%$) relative to incompatible trials ($2.09\%$). The main effect of task-order switch was not statistically significant, $F(1, 20) = 1.07$, $p = .313$, $\eta _p^2 = .05$. The interaction of compatibility in trial *N* and trial $N-1$ just missed the conventional level of statistical significance, $F(1, 20) = 4.22$, $p = .053$, $\eta _p^2 = .17$, although the BCE was slightly larger following compatible trials $N-1$ (4.88 percentage points) relative to incompatible trials $N-1$ (2.93 percentage points). Importantly, a sequential modulation was clearly present for task-order repetitions (4.53 percentage points), but almost absent for task-order switches ($-0.62$ percentage points), as indicated by a significant three-way interaction of trial *N* compatibility, trial $N-1$ compatibility, and task-order switch, $F(1, 20) = 10.33$, $p = .004$, $\eta _p^2 = .34$. The interaction of trial $N-1$ compatibility and task-order switch was also significant, $F(1, 20) = 9.60$, $p = .006$, $\eta _p^2 = .32$, with a larger difference between previously incompatible and compatible trials after task-order repetitions ($-1.73$ percentage points) relative to task-order switches (0.5 percentage points). Finally, the interaction of trial *N* compatibility and task-order switch missed statistical significance, $F(1, 20) = 3.90$, $p = .062$, $\eta _p^2 = .16$, although the BCE was slightly larger for task-order switches (5.06 percentage points) relative to task-order repetitions (2.75 percentage points).^5^

**TOS rates** Mean and individual TOS rates are visualized in Fig. 2E. The overall TOS rate of $$35.42\%$$ was statistically different from $$50\%$$, $$t(21) = -3.34$$, $$p = .003$$, $$d = -0.71$$. TOS rates were not significantly different between compatible ($$35.35\%$$) and incompatible ($$35.50\%$$) trials $$N-1$$, $$t(21) = -0.19$$, $$p = .852$$, $$d = -0.04$$, and the corresponding Bayes factor provided evidence for the absence of an effect, $$BF_{01} = 4.41 $$ $$(\pm 0.02\%)$$. When rerunning our analyses with wider priors, Bayes factors tended to be higher, reaching values of $$BF_{01} = 8.34$$ ($$\pm 0.13\%$$). Thus, the data of Experiment 1b support the independence of TOS rates from response conflict in the previous trial.

### Discussion

In Experiment 1b, participants indicated their task-order choice prior to each trial. A sequential modulation was descriptively observed for task-order repetition and switch trials for RTs. In contrast to Experiment 1a, however, this effect was stronger for task-order repetitions relative to switch trials. For PEs, the sequential modulation was only descriptively present for task-order repetition but not for task-order switch trials. Interestingly, despite a stronger separation of trials, and despite participants indicating which task they intend to perform first, compatibility in trial $N-1$ did not affect the choice to switch task order. In fact, the evidence for the independence of TOS rates from conflict in trial $N-1$ was even stronger for Experiment 1b as it was for Experiment 1a.

A point worth discussing is the statistical power obtained in both Experiment 1a and b, especially against the background of an indecisive result regarding the influence of trial $N-1$ compatibility on TOS rates in Experiment 1a. After excluding participants, our sample sizes for Experiment 1a and b were 20 and 22, respectively, which would imply that we can detect an effect size of at least $d = 0.66$ in the analyses of TOS rates with a power of $1-\beta = .8$ (Langenberg et al., 2023). Assuming a standard deviation of $\sigma = 21$ within each condition, together with a correlation of $\rho = .98$ between conditions, we can express the effect size $d = 0.66$ in terms of a detectable difference of about 3 percentage points.^6^ Thus, with the sample sizes in Experiments 1a and b, we can assume that the difference in TOS rates between compatible and incompatible trials $N-1$ is unlikely to be much higher than 3 percentage points, otherwise we likely would have detected a difference with frequentist inferential statistics. Nonetheless, to reduce the size of a detectable difference further, we increased the sample size in the following experiments.

**Fig. 3:**
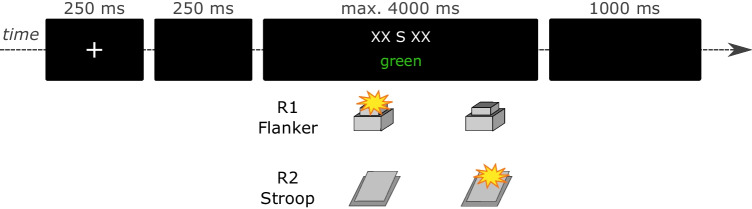
Trial procedure and tasks in Experiment 2a. *Note.* In the flanker task, participants responded to the identity of the central letter presented above screen center with manual key presses. In the Stroop task, participants responded to the color of the word presented below screen center with pedal presses. In this particular example, an S in the flanker task is flanked by the letters X and requires a manual response with the left key. The color word green in the Stroop task is written in green and requires a right pedal response (color words were presented originally in German). Thus, this example depicts a flanker incompatible, Stroop compatible, and response incompatible trial. Participants were free to perform either the manual task or the pedal task first in each trial. However, they were instructed to choose both possible task orders about equally often

Before we draw our final conclusions, we will report two further experiments, which conceptually extend the previous experiments in the following ways: First, in the previous experiments, stimuli for both tasks were presented in an integral manner (i.e., a single colored letter was associated with two tasks). We now separated the stimuli for each task in Experiments 2a and b to better segregate each task set (see also Ellenbogen and Meiran, 2011). This could be important, because separate stimuli increase the salience of task sets (and thus their task order), which could highlight a change in task order as a potential strategy in response to conflict (see also Dignath et al., 2015, Exp. 1). Second, we substituted the color and letter classification task with a flanker and a Stroop task from the conflict task literature (see Fig. 3). This implies that we have two ’types’ of conflict: Task-specific conflict which is unique to each subtask (i.e., the flanker or the Stroop task) and between-task response conflict which can arise when both tasks require incompatible responses (as in Experiment 1a and b). Note that in the following, we will refer to the compatibility of responses as ’response compatibility’. Further, we will deviate from the more common term of congruency in the context of conflict tasks, and instead will refer to trials in the Stroop or flanker task as either Stroop/flanker compatible or incompatible. This was done to avoid switching between the terms congruent/incongruent and compatible/incompatible, respectively, when describing the results.

## Experiment 2a

Experiment 2a is an extension of Experiment 1a, with the change that participants now responded to a Stroop and a flanker task, instead of to the color and letter task. Yet, as in Experiment 1a, task order was not announced in advance of a trial, but instead determined by the order of responses within a trial.

### Method

#### Participants

Forty-eight students (37 female) from the University of Tübingen, aged 18 to 34 years (M = 23.04 years, SD = 3.36) with the same criteria as in the previous experiments were recruited. Five participants had to be excluded during data pre-processing (see the Design and analysis section for further information).

#### Apparatus and stimuli

The computer and response devices were the same as in the previous experiments. Stimuli are illustrated in Fig. 3. The letters ‘S’ or ‘X’ presented in white above screen center served as (central) targets and flankers for the flanker task. Responses to the flanker task were given manually. The color words ’rot’ and ’grün’ (German for ’red’ and ’green’) presented in red or green color below screen center served as stimuli for the Stroop task. Responses to the Stroop task were given via a pedal presses.

#### Task and procedure

The trial sequence was identical to that of Experiment 1a and is illustrated in Fig. 3. After a short familiarization block, participants worked on fourteen experimental blocks of 48 trials each, resulting from three repetitions of all combinations of 4 flanker stimuli × 4 Stroop stimuli. Otherwise, the procedure was the same as in Experiments 1a and 1b.

**Fig. 4:**
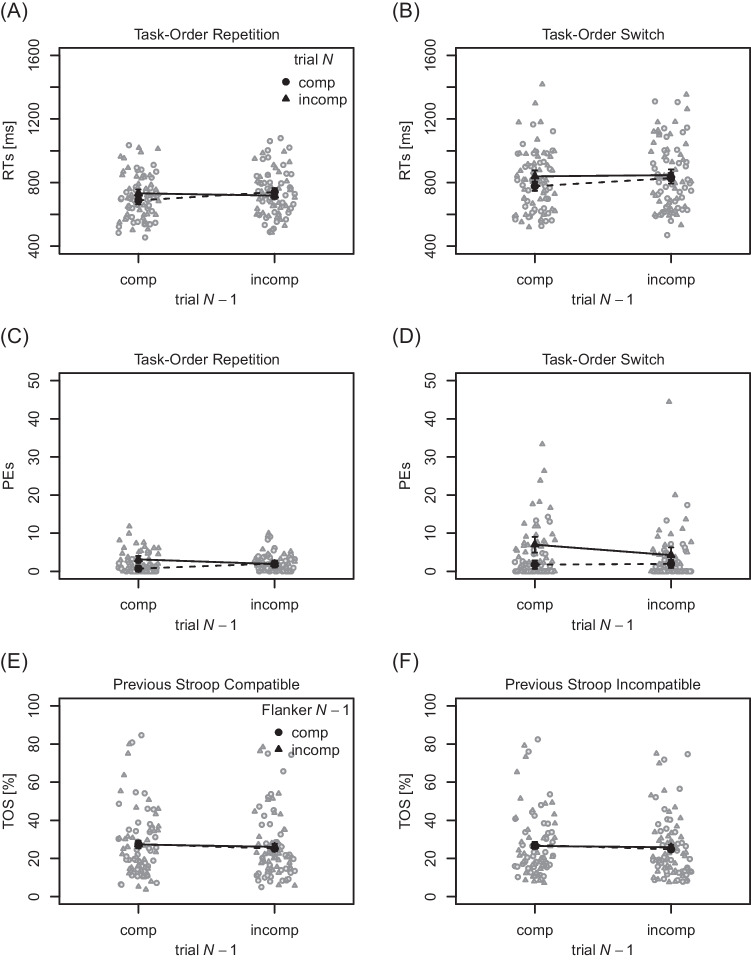
Mean response times, percentages error, and task-order switch rates for Experiment 2a. *Note.* (**A**) and (**B**) Results for response times (RTs) to the first performed task as a function of compatibility in trial *N* and compatibility in trial $N-1$, separately for task-order repetitions and switches, respectively. (**C**) and (**D**) Analogous results for percentages error (PEs). (**E**) and (**F**) Task-order switch (TOS) rates as a function of trial *N* - 1 compatibility (x-axis), trial *N* - 1 flanker compatibility (triangles and circles), and trial *N* - 1 Stroop compatibility (**E** and **F**). In all panels, *gray dots* and *triangles* reflect individual mean values. *Black dots* and *triangles* indicate mean values, aggregated across individual data points. *Error bars *around these values indicate $95\%$ confidence intervals after removing inter-subject variability (Morey, 2008); comp = compatible, incomp = incompatible

#### Design and analysis

A trial was considered flanker compatible when the target letter was the same as the flankers in the flanker task, otherwise it was considered flanker incompatible. A trial was considered Stroop compatible when the color matched the word meaning in the Stroop task, otherwise it was considered Stroop incompatible. Trials in which both responses were to-be-given on the same side were considered response compatible, and otherwise they were considered response incompatible. The main change relative to Experiment 1a was that the ANOVA on TOS rates included the factors trial $N-1$ response compatibility (incompatible vs. compatible), trial $N-1$ flanker compatibility (incompatible vs. compatible), and trial $N-1$ Stroop compatibility (incompatible vs. compatible). Bayes factors were calculated using the function *anovaBF* from the *BayesFactor* package (Morey & Rouder, 2022).^7^ Further, we ran two exploratory ANOVAs on RTs/PEs, each with either the factors trial *N* and trial $N-1$ flanker compatibility, or trial *N* and trial $N-1$ Stroop compatibility. The respective results are presented in Appendix A.

Data were pre-processed as in Experiment 1a: Trials with a general error in trial *N* were discarded in $1.45\%$ of the trials. Based on these data, two participants were excluded for having inter-response intervals of less than 50 ms in at least $20 \%$ of the trials. From the remaining data, $1.39\%$ were excluded because trial $N-1$ had a general error, and $3.92\%$ because trials *N* or trials $N-1$ had an inter-response interval of less than 50 ms. Based on these data, three participants were excluded for changing task order in less than $10\%$ of the times. Finally, $7.27\%$ of the trials with response errors in at least one of the tasks in trial $N-1$ were excluded for all analyses. For RT analysis, only correct trials *N* were considered (discarding $6.84\%$ of the trials) and $3.0\%$ of the trials were excluded as outliers. With the remaining 43 participants, we could detect an effect of at least $d = 0.44$ (or equivalently of $\eta _p^2 = .16$ in a repeated measures ANOVA with each factor having two levels; Langenberg et al., 2023) with a power of $1-\beta = .8$.

### Results

**RTs (response compatibility)** Mean and individual RTs to the first performed task are visualized in Fig. 4A and B. RTs were shorter for response compatible (758 ms) relative to incompatible (784 ms) trials *N*, $$F(1, 42) = 7.80$$, $$p = .008$$, $$\eta _p^2 = .16$$, thus a BCE was present. The main effect of response compatibility in trial $$N-1$$ reached statistical significance, $$F(1, 42) = 9.70$$, $$p = .003$$, $$\eta _p^2 = .19$$, with slightly longer RTs following response incompatible (783 ms) relative to compatible (759 ms) trials. The main effect of task-order switch was significant, $$F(1, 42) = 27.59$$, $$p < .001$$, $$\eta _p^2 = .40$$, with RTs being shorter when task order repeated (719 ms) than when it switched (823 ms). The interaction of compatibility in trial *N* and trial $$N-1$$ was significant, $$ F(1, 42) = 15.10$$, $$p < .001$$, $$\eta _p^2 = .26$$, indicating a sequential modulation. In particular, a BCE was present following compatible trials $$N-1$$ (53 ms), and it was almost absent following incompatible trials $$N-1$$ ($$-1$$ ms). Descriptively, this pattern was qualified by task order, with a numerically larger sequential modulation of the BCE after task-order repetitions (67 ms) than after switches (43 ms), although the corresponding three-way interaction did not reach statistical significance, $$F(1, 42) = 0.79$$, $$p = .379$$, $$\eta _p^2 = .02$$. Further, there was a tendency for a larger BCE after task-order switches (41 ms) relative to task-order repetitions (11 ms), which did not reach statistical significance, however, $$F(1,42) = 3.98$$, $$p = .053$$, $$\eta _p^2 = .09$$. The remaining interaction between trial $$N-1$$ compatibility and task-order switch was not statistically significant, $$F(1,42) = 0.51$$, $$p = .477$$, $$\eta _p^2 = .01$$.

**PEs (response compatibility)** Mean and individual PEs are visualized in Fig. 4C and D. PEs were lower for response compatible ($$1.62 \%$$) relative to incompatible ($$4.07 \%$$) trials *N*, $$F(1, 42) = 18.53$$, $$p < .001$$, $$\eta _p^2 = .31$$, thus a BCE was present in PEs. The main effect of response compatibility in trial $$N-1$$ was not statistically significant, $$F(1, 42) = 3.48$$, $$p = .069$$, $$\eta _p^2 = .08$$, although PEs were higher after response compatible ($$3.15\%$$) relative to incompatible trials ($$2.54\%$$). The main effect of task-order switch was statistically significant, $$F(1, 42) = 9.42$$, $$p = .004$$, $$\eta _p^2 = .18$$, with higher PEs after task-order switches ($$3.74 \%$$) relative to task-order repetitions ($$1.95 \%$$). The interaction of response compatibility in trial *N* and trial $$N-1$$ was significant, $$F(1, 42) = 13.07$$, $$p = .001$$, $$\eta _p^2 = .24$$, indicating a sequential modulation. The BCE was present following compatible trials $$N-1$$ (3.82 percentage points), and it was reduced following incompatible trials $$N-1$$ (1.08 percentage points). The interaction of trial *N* response compatibility and task-order switch was also statistically significant, $$ F(1, 42) = 5.51$$, $$p = .024$$, $$\eta _p^2 = .12$$, with a larger BCE after task-order switches (3.76 percentage points) relative to repetitions (1.14 percentage points). The interaction of trial $$N-1$$ response compatibility with task-order switch did not reach the conventional level of statistical significance, $$F(1, 42) = 3.88$$, $$p = .055$$, $$\eta _p^2 = .08$$, although the difference between previously compatible and incompatible trials $$N-1$$ was larger for task-order switches (1.26 percentage points) than task-order repetitions ($$-0.05$$ percentage points). Finally, the three-way interaction between trial *N* response compatibility, trial $$N-1$$ response compatibility, and task-order switch was not statistically significant, $$F(1,42) = 0.08$$, $$p = .779$$, $$\eta _p^2 <.01$$.^8^

**TOS rates** Mean and individual TOS rates as a function of trial $$N-1$$ response compatibility, trial $$N-1$$ flanker compatibility, and trial $$N-1$$ Stroop compatibility are visualized in Fig. 4E and F. The overall TOS rate of $$26.28\%$$ was significantly different from $$50\%$$, $$t(42) = -9.52$$, $$p < .001$$, $$d = -1.47$$. TOS rates were significantly different for previously response compatible ($$27.09\%$$) relative to previously response incompatible ($$25.47\%$$) trials $$N-1$$, $$F(1, 42) = 7.06, p = .011, \eta _p^2 = .14$$, aligning with a corresponding Bayes factor of $$BF_{01} = 0.12 $$ $$(\pm 3.53\%)$$. TOS rates did not statistically differ between previously flanker compatible ($$26.11\%$$) and previously flanker incompatible ($$26.45\%$$) trials $$N-1$$, $$F(1, 42) = 0.34, p = .565, \eta _p^2 = .01$$, and the corresponding Bayes factor was $$BF_{01} = 7.12 $$ $$(\pm 4.33\%)$$. TOS rates did also not statistically differ between previous Stroop compatible ($$26.52 \%$$) and previous Stroop incompatible ($$26.04 \%$$) trials $$N-1$$, $$F(1, 42) = 0.85, p = .361, \eta _p^2 = .02$$, and the corresponding Bayes factor was $$BF_{01} = 6.59 $$ $$(\pm 5.44\%)$$. None of the interactions reached statistical significance, $$F\text {s} \le 1.17, p\text {s} \ge .286, BF_{01}\text {s} \ge 3.11$$. When rerunning our analyses with wider priors, Bayes factors tended to be higher, giving a value of $$BF_{01} = 0.21 $$ $$(\pm 2.47 \%$$) for the main effect of trial $$N-1$$ response compatibility, and at least $$BF_{01} = 6.11$$ for all other main and interaction effects. In essence, the data of Experiment 2a suggest that TOS rates are lower after response incompatible relative to response compatible trials, although they seem to be independent from conflict in each subtask.

### Discussion

In Experiment 2a, a flanker and a Stroop task served as the subtasks of a dual-task. When analyzing the influence of response conflict and task order on RTs and PEs, we observed a BCE and its sequential modulation. Additionally, this sequential modulation was at least descriptively smaller for task-order switches relative to task-order repetitions in RTs. Regarding the role of conflict influencing mechanisms of task selection, we did not obtain evidence suggesting that conflict within each subtask influences the chosen order of tasks in the following trial. Interestingly though, participants were less likely to change their task order after response incompatible relative to compatible trials, matching the descriptive trend observed in Experiment 1a. However, as in Experiment 1a, this difference of 1.62 percentage points was relatively small in its absolute magnitude.

## Experiment 2b

Similar to how Experiment 2a extended Experiment 1a, Experiment 2b extends Experiment 1b. Thus, in Experiment 2b, participants responded to a flanker and a Stroop task (see Fig. 3), but they announced their task order prior to a trial (as was done in Experiment 1b).

### Method

#### Participants

Forty-eight students (40 female) from the University of Bremen, aged 20 to 47 years (M = 25.33 years, SD = 5.53) were recruited. Ten participants had to be excluded during data pre-processing (see Design and Analysis for further information).

#### Apparatus, stimuli, task, and procedure

Apparatus, stimuli, tasks, trial numbers, and block numbers were the same as in Experiment 2a. The trial procedure was similar to that of Experiment 1b. That is, upon the presentation of a white "?", participants indicated their task order via two simultaneous presses of the manual or pedal keys. After the task order was registered with the onset of the simultaneous keys presses, a blank screen was shown for 1500 ms, and the trial proceeded as in Experiment 2a.

#### Design and analysis

Data analyses were identical to those of Experiment 2a (the exploratory ANOVAs on RTs/PEs for each subtask can be found in Appendix B). Data pre-processing were as in the previous experiments: After excluding the familiarization block and after coding choice RTs shorter than 100 ms or longer than 4000 ms as a ’general error’, we had to exclude six participants for committing an error or general error in at least $20 \%$ of the trials. Afterwards, trials with a general error in trial *N* were discarded for the remaining participants ($6.29\%$). Next, $5.94\%$ of trials were excluded because trial $N-1$ had a general error, and $0.53\%$ because trials *N* or trials $N-1$ had an inter-response interval of less than 50 ms. Based on the remaining data, four participants were excluded for changing task order in less than $10\%$ of the times. Finally, $4.35\%$ of the trials with response errors in at least one of the tasks in trial $N-1$ were excluded for all analyses. For RT analysis, only correct trials *N* were considered (discarding $4.24\%$ of the trials) and $3.13\%$ of the trials were excluded as outliers. With the remaining 38 participants, we could detect an effect of at least $d = 0.47$ (or equivalently $\eta _p^2 = .18$) with a power of $1-\beta = .8$.

### Results

**Fig. 5:**
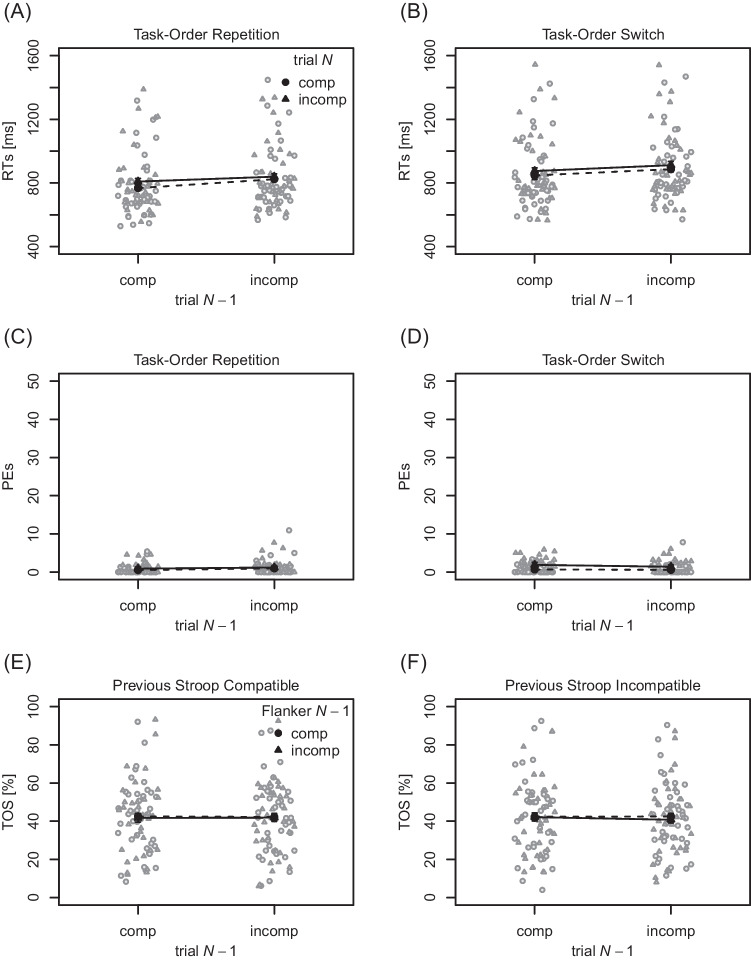
Mean response times, percentages error, and task-order switch rates for Experiment 2b. *Note.* (**A**) and (**B**) Results for response times (RTs) to the first performed task as a function of compatibility in trial *N* and compatibility in trial $N-1$, separately for task-order repetitions and switches, respectively. (**C**) and (**D**) Analogous results for percentages error (PEs). (**E**) and (**F**) Task-order switch (TOS) rates as a function of trial *N* - 1 compatibility (x-axis), trial *N* - 1 flanker compatibility (triangles and circles), and trial *N* - 1 Stroop compatibility (**E** and **F**). In all panels, *gray dots *and *triangles* reflect individual mean values. *Black dots *and *triangles* indicate mean values, aggregated across individual data points. *Error bars* around these values indicate $95\%$ confidence intervals after removing inter-subject variability (Morey, 2008); comp = compatible, incomp = incompatible

**RTs (response compatibility)** Mean and individual RTs to the first performed task are visualized in Fig. 5A and B. RTs were shorter for response compatible (831 ms) relative to incompatible (859 ms) trials *N*, $F(1, 37) = 13.99$, $p = .001$, $\eta _p^2 = .27$, thus a BCE was present. The main effect of response compatibility in trial $N-1$ reached statistical significance, $F(1, 37) = 44.81$, $p < .001$, $\eta _p^2 = .55$, with longer RTs following response incompatible (865 ms) relative to compatible (825 ms) trials. The main effect of task-order switch was significant, $F(1, 37) = 57.50$, $p < .001$, $\eta _p^2 = .61$, with RTs being shorter when task order repeated (810 ms) than when it switched (880 ms). Descriptively, the BCE was slightly larger following compatible trials $N-1$ (34 ms) than incompatible trials $N-1$ (20 ms). Yet, in contrast to the previous experiments, the interaction of compatibility in trial *N* and trial $N-1$ was not statistically significant, $F(1, 37) = 1.81$, $p = .187$, $\eta _p^2 = .05$, indicating a potential absence of a sequential modulation. Additionally, none of the other interactions were statistically significant; trial *N* response compatibility $\times$ order switch, $F(1, 37) = 0.03$, $p = .871$, $\eta _p^2 < .01$; trial $N-1$ response compatibility $\times$ order switch, $F(1, 37) = 0.12$, $p = .730$, $\eta _p^2 < .01$; trial *N* response compatibility $\times$ trial $N-1$ response compatibility $\times$ order switch, $F(1, 37) = 1.02$, $p = .319$, $\eta _p^2 = .03$. Yet, the sequential modulation was descriptively larger for task-order repetitions (25 ms) than for task-order switches (3 ms).

**PEs (response compatibility)** Mean and individual PEs are visualized in Fig. 5C and D and were generally rather low. PEs were lower for response compatible ($0.69\%$) relative to incompatible ($1.35\%$) trials *N*, $F(1, 37) = 15.42$, $p < .001$, $\eta _p^2 = .29$, thus a BCE was present in PEs. The main effect of response compatibility in trial $N-1$ was not statistically significant, $F(1, 37) = 0.02$, $p = .879$, $\eta _p^2 < .01$, and PEs were only barely higher after response incompatible ($1.03\%$) relative to compatible trials ($1.00\%$). The main effect of task-order switch was not statistically significant, $F(1, 37) = 1.30$, $p = .262$, $\eta _p^2 = .03$, with descriptively higher PEs after task-order switches ($1.13\%$) relative to task-order repetitions ($0.9\%$). As with RTs, the interaction of response compatibility in trial *N* and trial $N-1$ was not statistically significant, $F(1, 37) = 1.31$, $p = .260$, $\eta _p^2 = .03$, indicating a potentially absent sequential modulation. Descriptively, the BCE was marginally larger following compatible than incompatible trials $N-1$ (0.80 and 0.51 percentage points, respectively). The interaction of trial *N* response compatibility and task-order switch was statistically significant, $F(1, 37) = 4.35$, $p = .044$, $\eta _p^2 = .11$, with a slightly larger BCE after task-order switches (1.01 percentage points) relative to repetitions (0.3 percentage points). The interaction of trial $N-1$ response compatibility with task-order switch did also reach statistical significance, $F(1, 37) = 7.00$, $p = .012$, $\eta _p^2 = .16$, indicating a larger difference between previously compatible and incompatible trials $N-1$ for task-order repetitions (0.36 percentage points) than task-order switches ($-0.31$ percentage points). Finally, the three-way interaction between trial *N* response compatibility, trial $N-1$ response compatibility, and task-order switch was not statistically significant, $F(1,37) = 0.15$, $p = .701$, $\eta _p^2 <.01$.

**Fig. 6:**
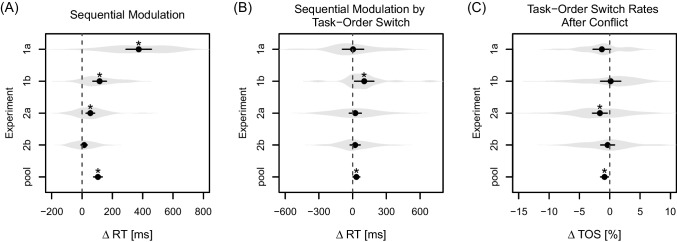
Summary of the effects relevant to the present study. *Note.* In each panel, we show an effect relevant to the present study as a difference variable (of differences; see Langenberg et al. 2023) separately for each experiment and when pooling the data (’pool’) from all experiments. *Gray shaded areas* indicate kernel density estimates across individual data. *Dots* and *lines* indicate the mean difference with a corresponding 95% confidence interval. A ’*’ above a mean indicates that this mean is significantly different from zero. **A** The sequential modulation of the BCE, that is, the two-way interaction between trial *N* compatibility and trial $N-1$ compatibility. Values were calculated by first averaging across task order and then contrasting the BCE for compatible and incompatible trials $N-1$. **B** The variation of the sequential modulation by task-order switch, that is, the three-way interaction between trial *N* compatibility, trial $N-1$ compatibility, and task-order switch. Values were computed by contrasting the sequential modulation for task-order repetitions and switches. **C** The influence of conflict in trial $N-1$ on the tendency to switch task order. Values were calculated by contrasting task-order switch rates for incompatible and compatible trials $N-1$ (averaged across trial $N-1$ flanker and Stroop compatibility for Experiments 2a and 2b)

**TOS rates** Mean and individual TOS rates as a function of trial $N-1$ response compatibility, trial $N-1$ flanker compatibility, and trial $N-1$ Stroop compatibility are visualized in Fig. 5E and F. The overall TOS rate of $42\%$ was significantly different from $50\%$, $t(37) = -2.65$, $p = .012$, $d = -0.44$. TOS rates did not significantly differ between previously response compatible ($42.18\%$) and response incompatible ($41.83\%$) trials $N-1$, $F(1, 37) = 0.38, p = .543, \eta _p^2 = .01$, aligning with a corresponding Bayes factor of $BF_{01} = 9.30$ $(\pm 5.3\%)$. TOS rates did also not statistically differ between previously flanker compatible ($42.39\%$) and previously flanker incompatible ($41.62\%$) trials $N-1$, $F(1, 37) = 2.03, p = .163, \eta _p^2 = .05$, and the corresponding Bayes factor was $BF_{01} = 3.99$ $(\pm 5.57\%)$. TOS rates did also not statistically differ between previous Stroop compatible ($42.08 \%$) and previous Stroop incompatible ($41.93 \%$) trials $N-1$, $F(1, 37) = 0.07, p = .797, \eta _p^2 < .01$, and the corresponding Bayes factor was $BF_{01} = 8.20$ $(\pm 8.28\%)$. None of the interactions reached statistical significance, $F\text {s} \le 0.49, p\text {s} \ge .487, BF_{01}\text {s} \ge 3.22$. When rerunning our analyses with wider priors, Bayes factors tended to be higher, reaching values of at least $BF_{01} > 5.64$ for any of the main or interaction effects.

### Discussion

In Experiment 2b, a flanker and a Stroop task served as the subtasks of a dual-task and participants announced their responses prior to a trial. When analyzing the influence of response conflict and task order on RTs and PEs, we observed a BCE but no statistically significant sequential modulation. Descriptively though, the BCE was smaller after response incompatible than after compatible trials, and the sequential modulation was larger for task-order repetitions than for switches. Yet, since the respective effects were not statistically significant, their interpretation must be treated with care. Regarding the role of conflict influencing mechanisms of task selection, we did not obtain evidence suggesting that conflict within each subtasks influences the chosen order of tasks in the following trial. Additionally, and in contrast to Experiment 2a, there was again no influence of response conflict on task order, matching the results of Experiment 1b.

## General discussion

The present study investigated the interaction of between-task response conflict during task performance and task organization processes during task selection in dual-task experiments. In particular, we investigated whether the efficiency of cognitive control in a dual-task depends on the repetition or alternation of the task order, as indexed by different sizes of the sequential modulation of the BCE. Further, we tested whether response conflicts at a lower level of task performance not only prompt adaptation mechanisms at the same level, but also at a higher level of task selection.

In four experiments, participants were asked to complete two (sub)tasks on each trial, but were free to choose the order of these tasks. In Experiment 1a, the task-order choice decision was apparent from the temporal organization of the response onsets. In Experiment 1b, processes of task choice and task performance were separated by instructing participants to announce their task order before a trial. Experiments 2a and 2b extended Experiment 1a and 1b, respectively, by separating the stimuli for each task. For this purpose, the subtasks from Experiments 1a and 1b were replaced by a flanker and a Stroop task.

Because our results with respect to our main questions varied across the different experiments in terms of their descriptive trend and in terms of their statistical significance, we present them here jointly once more within a single plot. In particular, Fig. 6 depicts (A) the efficiency of conflict resolution in terms of a sequential modulation of the BCE, (B) its variation as a function of task-order repetitions versus switches, and (C) the influence of previously experienced conflict on the decision about a task-order switch, for each experiment. All panels also present the respective effect when pooling the data across all the experiments.

First we replicated that the BCE is subject to a sequential modulation (e.g., Durst & Janczyk, 2019; Janczyk, 2016; Renas et al., 2018; Scherbaum et al., 2015; Schonard et al., 2020) in three out of four experiments. On average, the BCE was larger following R1-R2 compatible trials, whereas it was reduced and sometimes even reversed following incompatible trials. Interestingly, this sequential modulation was not significant in Experiment 2b, where participants announced their task-order choice in advance and where they worked on a flanker and a Stroop task. Currently, we lack a final explanation for this finding. However, one aspect that might have contributed to the non-significant result is that the sequential modulation seems to be sensitive to the way trials are separated. From Fig. 6A it appears that announcing the task order (Experiments 1a vs. 1b), reduces the strength of the sequential modulation, so that a generally less pronounced sequential modulation with separated tasks (Experiments 2a and 2b) may then turn out non-significantly (Experiment 2b). In hindsight, a less pronounced sequential modulation when separating task order selection and task performance is not surprising, because deciding which task to select increases the time between trials. Studies in the single-task context have suggested that in specific situations, the sequential modulation of conflict effects decreases with longer temporal separation between trials (Egner et al., 2010; but see Schiltenwolf, Kiesel, Frings et al., 2023, Schiltenwolf, Kiesel & Dignath, 2023, for evidence demonstrating the temporal stability of control states). Additionally, already Gratton et al. (1992) has argued that conflict adaptation may be driven by expectations. In particular, it has been demonstrated in single-task studies that participants expect the same trial type to repeat (Duthoo et al., 2013), which facilitates the retrieval of a previously recruited control state in order to prepare for the upcoming trial (see Egner, 2014, for a more detailed discussion). It thus could be that participants in Experiments 1b and 2b were more focused on deriving a deliberate decision about the task order than to prepare for the upcoming trial type.

An interesting and novel observation from Experiment 1b is that the sequential modulation of the BCE is slightly less pronounced after task-order switches than after repetitions. This is intriguing, because subtasks, stimuli, and responses are identical for task-order switches and repetitions, making it unlikely that some low-level property of a trial mediates this effect. Yet, it is difficult to interpret this effect because there was no variation in sequential modulation with task-order repetitions/switches in Experiment 1a, and in Experiments 2a and 2b the effect was descriptively present but not statistically significant. When pooling the data across experiments, the effect was small, but statistically significant ($95\%$ confidence interval: [3 ms, 65 ms]; see Fig. 6B). Clearly, any interpretation of this relatively small and ’barely significant result’ should be treated with great caution, especially when considering that pooling the data is a rough way of estimating a mean effect. Nevertheless, reviewing the possible underlying mechanisms may come with theoretical value. As discussed in the Introduction, a most straightforward explanation is to assume that the task-order set as a higher-order representation is also stored within the so-called episodic file (Braem et al., 2014; Dignath et al., 2019; Egner, 2014; Kreutzfeldt et al., 2016; Spapé & Hommel, 2008). Specifically, when task order repeats, the episodic file formed in the previous trial can be retrieved, and its associated cognitive control state re-instantiated. This recruitment of cognitive control during a conflict-prone incompatible trial can mitigate the negative impact of between-task response conflict in the subsequent trial. If task order changes, however, the previous association between cognitive control and task order may be weakened, leading to a disruption in attentional recruitment. Such an interpretation would be in line with a hierarchical view of dual-tasking (e.g., Logan & Gordon, 2001), where lower levels responsible for task performance receive input from higher levels of task selection, and their associated control states. It also aligns somewhat with the separated order set hypothesis, which posits that task order is a distinct representation, stored separately from the specific information about subtasks (e.g., Kübler et al., 2022a, see also Stelzel et al., 2008; Szameitat et al. 2006).

Our result with respect to a maybe reduced efficiency of conflict resolution after task-order switches are further intriguing, since research on context-specific proportion congruency manipulations demonstrated that attentional control states are robust to changes in particular stimuli, responses, or even task sets (Surrey et al., 2017), matching the generally small influence of task-order decisions in this present study. Yet, given that our results were heterogeneous, it remains open to future research to explore whether the reduced efficiency of conflict resolution after task-order switches can be replicated, and if at all, under which circumstances. But investigating this phenomenon might provide valuable insights into the way our cognitive system is organized when we deal with multiple simultaneous task requirements.

A third finding is a slight tendency for repeating task order after response incompatible relative to response compatible trials in Experiments 1a and 2a, although such an effect was absent when separating the processes of task order and task performance in Experiments 1b and 2b. When pooling the data across experiments, this bias turned out statistically significant ($95\%$ confidence interval: $[-1.52, \; -0.20]$ percentage points; see Fig. 6C). Thus, from a statistical standpoint, we have no evidence for the idea that participants in a dual-task voluntarily change task order after conflict. This clearly argues against a motivational account in which participants explore the possibility of changing the task order as a way of escaping from a conflict-associated situation (Dignath et al., 2015). Interestingly, however, the general tendency to repeat task order after conflict implies that individuals have a tendency to switch the individual subtasks after conflict (i.e., repeating task order implies constantly switching between individual subtasks). Thus, perhaps our results are not as contradictory to those of Dignath et al. (2015) as they first seem. Yet, an alternative and maybe more intuitive interpretation of the tendency to repeat task order after conflict, is to assume that conflict strengthens currently active task representations (see also Verguts and Notebaert, 2008), including the task-order set. According to the availability heuristic, this would then bias participants to repeat the same task order in the next trial (i.e., to keep the same task order for the upcoming trial). However, whether significant or not when pooling the data, the tendency to repeat task order after conflict was rather small in its absolute values, ranging from 0.15 to $-1.62$ percentage points across all experiments. Thus, it is unlikely that this effect is of large practical importance, leading us to conclude that higher-order processes of task-order selection at the one hand are practically independent from conflict signals at a lower level of task performance. Such an independence would then again align with results indicating that task-order sets do not contain specific S-R relations constituting their subtasks (Kübler et al., 2022a).

A last finding, which we did not aim at investigating in the first place though, is a strong bias to repeat the same task order across successive trials. More specifically, across all experiments, only $26\%$ to $42\%$ of the trials were task-order switches. This observation is not entirely new. Indeed, a task order repetition bias was previously reported by Kübler et al. (2018; see also De Jong, 1995, Exp. 2). In this study, a subgroup of participants could decide which task to perform first, but had no control over stimulus presentation. Instead, stimuli for both tasks were presented randomly, separated by a stimulus-onset-asynchrony. The present study replicates the result of Kübler et al. and extends it to dual-tasks without a stimulus-onset-asynchrony. The observation of a clear task-order repetition bias is particularly interesting when interpreted against the background of previous studies on voluntary task switching (Arrington & Logan, 2004, 2005). Here, a consistent finding is that individuals tend to repeat the same task. In contrast, the present research found the opposite pattern, with more switches for individual subtasks (i.e., more task-order repetitions). Yet, such a switch bias for individual subtasks (i.e., a repetition bias for task order) is well in line with research on task-order control (e.g., De Jong, 1995; Luria & Meiran, 2003, 2006). In particular, as mentioned in the Introduction, changing task order requires alternating the task-order set, and is thus detrimental to performance. Therefore, the present results provide further evidence for such a task-order representation which biases task-order choices. Furthermore, the present results fit nicely with the expected value of control model by Shenhav et al. (2013), which states that agents anticipate upcoming control demands and weigh them against potential benefits (e.g., rewards to be obtained) or costs (e.g., effort required to exert control). Arguably, repeating task order has a higher utility, because task-order switch costs can be avoided.

In summary, this study was motivated by a hierarchical perspective on multitasking, which posits the existence of various conflict-control loops at different levels (Schuch et al., 2019). Specifically, our aim was to investigate how task selection influences task performance, and more importantly, how between-task response conflict during task performance impacts task selection. We observed a tendency for cognitive control to be less efficient after task-order switches compared to repetitions. This latter effect may suggest that modifying higher-order representations, which guide lower-level task performance, can interfere with the processes of conflict resolution at these lower levels. However, since this effect was only clearly observed in one out of four experiments (Exp. 1b), further research is needed to evaluate its reliability. Further, in two out of four experiments, we noticed a slight tendency to repeat task order after encountering conflict. This suggests that conflict at a lower level of task performance may prompt adaptations at a higher level of task selection. However, this effect was relatively small and only reached statistical significance in Experiment 2a. Thus, while conflict at a lower level of task performance may signal adaptation processes at the same level, as suggested by the sequential modulation of the BCE, this conflict signal is likely to be function-specific and may not strongly influence the level of task-order control.

## Figures and Tables

**Fig. 7:**
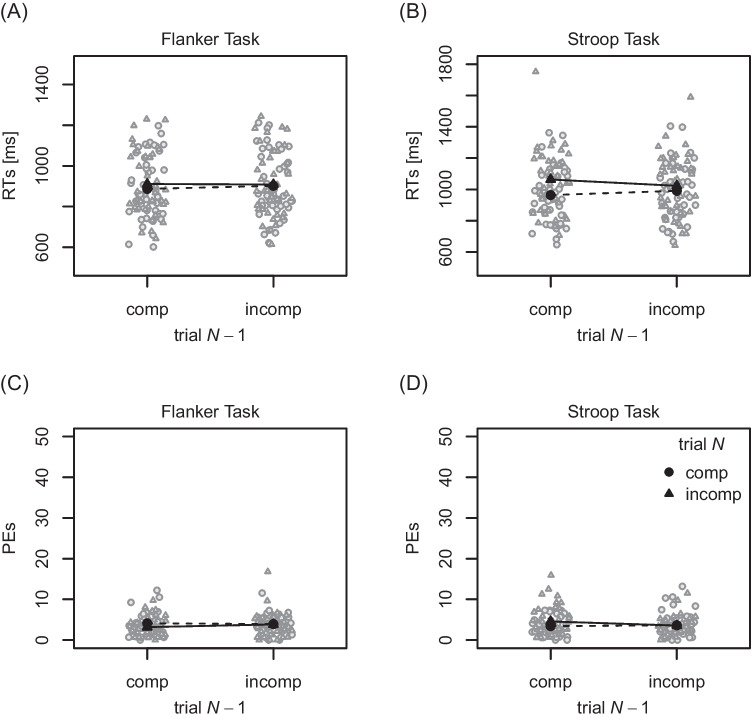
Response times and percentages error for the flanker and Stroop subtasks in Experiment 2a. *Note.* (**A**) and (**B**) Results for response times (RTs) as a function of trial *N* and trial $N-1$ compatibility, separately for the flanker and Stroop task, respectively. (**C**) and (**D**) The analogous results for percentages error (PEs). In all panels, *gray dots* and *triangles* reflect individual mean values for compatible and incompatible trials *N*, respectively. *Black dots* and *triangles* indicate mean values, aggregated across individual data points. *Error bars *around these values indicate $95\%$ confidence intervals after removing inter-subject variability (Morey, 2008)

**Fig. 8:**
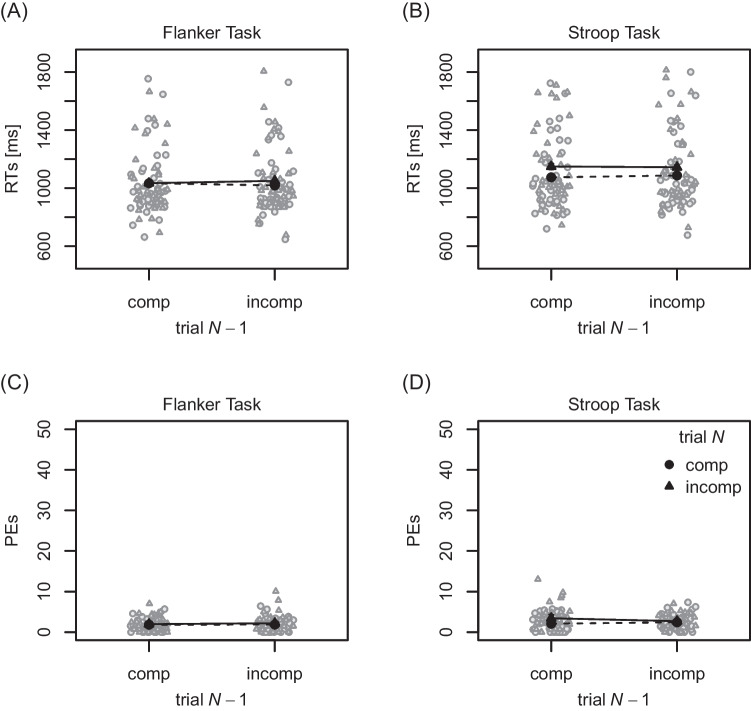
Response times and percentages error for the flanker and Stroop subtasks in Experiment 2b. *Note.* (**A**) and (**B**) Results for response times (RTs) as a function of trial *N* and trial $N-1$ compatibility, separately for the flanker and Stroop task, respectively. (**C**) and (**D**) The analogous results for percentages error (PEs). In all panels, *gray dots* and *triangles* reflect individual mean values for compatible and incompatible trials *N*, respectively. *Black dots* and *triangles* indicate mean values, aggregated across individual data points. *Error bars* around these values indicate $95\%$ confidence intervals after removing inter-subject variability (Morey, 2008)

## Exploratory analyses for the flanker and Stroop task of Experiment 2a

In this Appendix, we present the exploratory analyses for trial *N* flanker compatibility $\times$ trial $N-1$ flanker compatibility and for trial *N* Stroop compatibility $\times$ trial $N-1$ Stroop compatibility.

**RTs for the flanker subtask** Mean and individual RTs to the flanker subtask are visualized in Fig. 7A. RTs ($2.4\%$ trials excluded as outliers) were shorter for flanker compatible (895 ms) relative to flanker incompatible (910 ms) trials *N*, $F(1, 42) = 10.18$, $p = .003$, $\eta _p^2 = .20$, thus a flanker effect was present. The main effect of flanker compatibility in trial $N-1$ was not statistically significant, $F(1, 42) = 1.63$, $p = .208$, $\eta _p^2 = .04$, but RTs were descriptively slightly longer following flanker incompatible (905 ms) than flanker compatible (900 ms) trials. The interaction of flanker compatibility in trial *N* and trial $N-1$ just missed the conventional level of significance, $F(1, 42) = 3.60$, $p = .065$, $\eta _p^2 = .08$, although the flanker effect was larger after compatible (24 ms) than incompatible trials (7 ms).

**PEs for the flanker subtask** Mean and individual PEs of the flanker subtask are visualized in Fig. 7C. PEs were descriptively higher for flanker compatible ($4.01 \%$) relative to flanker incompatible ($3.49 \%$) trials *N*, but this difference was not statistically significant, $F(1, 42) = 2.68$, $p = .109$, $\eta _p^2 = .06$. The main effect of flanker compatibility in trial $N-1$ was also not statistically significant, $F(1, 42) = 1.20$, $p = .280$, $\eta _p^2 = .03$, although PEs were slightly higher following flanker incompatible ($3.87 \%$) than flanker compatible ($3.64 \%$) trials. The interaction of flanker compatibility in trial *N* and trial $N-1$ reached the conventional level of significance, $F(1, 42) = 4.63$, $p = .037$, $\eta _p^2 = .10$, with a higher reversed flanker effect after compatible ($-0.93$ percentage points) than incompatible trials ($-0.09$ percentage points).

**RTs for the Stroop subtask** Mean and individual RTs to the Stroop subtask are visualized in Fig. 7B. RTs ($2.82\%$ trials excluded as outliers) were shorter for Stroop compatible (977 ms) relative to Stroop incompatible (1043 ms) trials *N*, $F(1, 42) = 50.11$, $p < .001$, $\eta _p^2 = .54$, thus a Stroop effect was present. The main effect of Stroop compatibility in trial $N-1$ was not statistically significant, $F(1, 42) = 1.37$, $p = .249$, $\eta _p^2 = .03$, although RTs were slightly longer following Stroop compatible (1013 ms) than Stroop incompatible (1007 ms) trials. The interaction of Stroop compatibility in trial *N* and trial $N-1$ was statistically significant, $F(1, 42) = 32.21, p < .001, \eta _p^2 = .43$, with a larger Stroop effect after compatible (99 ms) than incompatible trials (32 ms).

**PEs for the Stroop subtask** Mean and individual PEs of the Stroop subtask are visualized in Fig. 7D. PEs were higher for Stroop incompatible ($4.06 \%$) relative to Stroop compatible ($3.52 \%$) trials *N*, $F(1, 42) = 4.13$, $p = .048$, $\eta _p^2 = .09$. The main effect of Stroop compatibility in trial $N-1$ was statistically significant, $F(1, 42) = 4.41$, $p = .042$, $\eta _p^2 = .10$, with higher PEs following Stroop compatible ($3.99 \%$) than Stroop incompatible ($3.59 \%$) trials. The interaction of Stroop compatibility in trial *N* and trial $N-1$ did not reach the conventional level of significance, $F(1, 42) = 2.53$, $p = .119$, $\eta _p^2 = .06$, although the Stroop effect was slightly larger after compatible (1.15 percentage points) than incompatible trials ($-0.06$ percentage points).

## Exploratory analyses for the flanker and Stroop task of Experiment 2b

In this Appendix, we present the exploratory analyses for trial *N* flanker compatibility $\times$ trial $N-1$ flanker compatibility and for trial *N* Stroop compatibility $\times$ trial $N-1$ Stroop compatibility.

**RTs for the flanker subtask** Mean and individual RTs to the flanker subtask are visualized in Fig. 8A. RTs ($2.42\%$ trials excluded as outliers) were shorter for flanker compatible (1026 ms) relative to flanker incompatible (1042 ms) trials *N*, $F(1, 37) = 7.40$, $p = .010$, $\eta _p^2 = .17$, thus a flanker effect was present. The main effect of flanker compatibility in trial $N-1$ was not statistically significant, $F(1, 37) = .003$, $p = .859$, $\eta _p^2 < .01$, and RTs were only slightly longer following flanker incompatible (1035 ms) than flanker compatible (1034 ms) trials. The interaction of flanker compatibility in trial *N* and trial $N-1$ was statistically significant, $F(1, 37) = 4.93$, $p = .033$, $\eta _p^2 = .12$. Surprisingly, however, the flanker effect was larger after incompatible (31 ms) than after compatible trials (1 ms).

**PEs for the flanker subtask** Mean and individual PEs of the flanker subtask are visualized in Fig. 8C. PEs were higher for flanker incompatible ($2.10 \%$) relative to flanker compatible ($1.86 \%$) trials *N*, although the corresponding main effect was not statistically significant, $F(1, 37) = 2.23$, $p = .144$, $\eta _p^2 = .06$. The main effect of flanker compatibility in trial $N-1$ was also not statistically significant, $F(1, 37) = 0.39$, $p = .538$, $\eta _p^2 = .01$, and PEs were only slightly higher following flanker incompatible ($2.04 \%$) than flanker compatible ($1.92 \%$) trials. The interaction of flanker compatibility in trial *N* and trial $N-1$ was not significant, $F(1, 37) = 0.23$, $p = .636$, $\eta _p^2 = .01$, with a slightly larger flanker effect after incompatible (0.32 percentage points) than compatible trials (0.15 percentage points).

**RTs for the Stroop subtask** Mean and individual RTs to the Stroop subtask are visualized in Fig. 8B. RTs ($2.61\%$ trials excluded as outliers) were shorter for Stroop compatible (11081 ms) relative to Stroop incompatible (1147 ms) trials *N*, $F(1, 37) = 43.00$, $p < .001$, $\eta _p^2 = .54$, thus a Stroop effect was present. The main effect of Stroop compatibility in trial $N-1$ was not statistically significant, $F(1, 37) = 0.41$, $p = .526$, $\eta _p^2 = .01$, and RTs were only slightly shorter following Stroop compatible (1112 ms) than Stroop incompatible (1116 ms) trials. The interaction of Stroop compatibility in trial *N* and trial $N-1$ was not statistically significant, $F(1, 42) = 1.69$, $p = .202$, $\eta _p^2 = .04$, with a descriptively larger Stroop effect after compatible (75 ms) than incompatible trials (57 ms).

**PEs for the Stroop subtask** Mean and individual PEs of the Stroop subtask are visualized in Fig. 8D. PEs were higher for Stroop incompatible ($3.08 \%$) relative to Stroop compatible ($2.26 \%$) trials *N*, $F(1, 37) = 4.79$, $p = .035$, $\eta _p^2 = .11$. The main effect of Stroop compatibility in trial $N-1$ was not statistically significant, $F(1, 37) = 0.48$, $p = .494$, $\eta _p^2 = .01$, with descriptively higher PEs following Stroop compatible ($2.77 \%$) than Stroop incompatible ($2.57 \%$) trials. The interaction of Stroop compatibility in trial *N* and trial $N-1$ reached the conventional level of significance, $F(1, 37) = 4.30$, $p = .045$, $\eta _p^2 = .10$, with the Stroop effect being slightly larger after compatible (1.35 percentage points) than incompatible trials (0.28 percentage points).

## Footnotes

1 Note that Experiment 2b was conducted after an anonymous reviewer suggested that the way task-order selection was established and the way the dual-task stimuli were presented should be fully crossed across experiments.

2 Trial numbers were rather small for the RT and PE analyses, as participants tended to repeat their task order. For Experiment 1a, the first and last quartiles for the distribution of trial numbers in each condition of trial *N* $\times$ trial $N-1$ compatibility entering the analysis of RTs after a task-order switch were $Q_{25\%} = 15$ and $Q_{75\%} = 47$, respectively.

3 Because Pieczykolan and Huestegge (2019) previously reported an influence of trial *N* compatibility on the scheduling of task order, we also exploratorily submitted TOS rates to an ANOVA with both the factors of trial *N* and trial $N-1$ compatibility. However, in this analysis, neither of the main effects nor the interaction turned out statistically significant, trial *N* compatibility, $F(1, 19) = 0.04$, $p = .849$, $\eta _p^2 < .01$; trial $N-1$ compatibility, $F(1, 19) = 3.49$, $p = .077$, $\eta _p^2 = .16$; interaction, $F(1, 19) = 2.38$, $p = .139$, $\eta _p^2 = .11$.

4 Note that in case participants did not respond in accordance with their announced task order, the respective trial was considered a ’general error’. Additionally, we coded task-order choice RTs smaller than 100 ms or larger than 4000 ms as a ’general error’.

5 When taking a closer look at Fig. 2D, one might suspect the data point on the upper right to be an influential outlier. We thus reran the analysis of PEs after (entirely) excluding the respective participant, which did not change the overall conclusions. Yet, in this case the main effect of trial $N\!-\!1$ compatibility and the interaction of trial *N* compatibility with trial $N\!-\!1$ compatibility turned out significant, $F(1, 19) \!=\! 4.93$, $p \!=\! .039$, $\eta _p^2 \!=\! .21$, and, $F(1, 19) \!=\! 9.71$, $p \!=\! .006$, $\eta _p^2 \!=\! .34$, respectively.

6 The values for $\sigma$ and $\rho$ are based on the average standard deviation within and correlation between conditions of Experiments 1a and 1b. The detectable difference of $\mu _X$ was calculated by rearranging the equation $d = \frac{\mu _X}{\sigma _X}$, with $\sigma _X = \sqrt{2\cdot \sigma ^2 (1-\rho )}$ (see Langenberg et al. 2023).

7 Note that there has been recent critique on the way Bayesian ANOVAs are implemented (van den Bergh et al., 2023; Singmann et al., 2023). To this end, we will remain a focus on the classical frequentist ANOVA. In addition, we reran our analyses with JASP (JASP Team, 2023, v. 0.17.3.0) to accommodate for some of the shortcomings of the *anovaBF* function (van den Bergh et al., 2023). This left our conclusions unchanged, however.

8 When taking a closer look at Fig. 4D, one might suspect the data point on the upper right to be an influential outlier. To this end, we reran the analysis of PEs after (entirely) excluding the respective participant, which did not change the overall conclusions. Yet, in this case the main effect of trial $N-1$ compatibility and the interaction of trial $N-1$ compatibility with task-order switch turned out significant, $F(1, 41) = 5.11$, $p = .029$, $\eta _p^2 = .11$, and, $F(1, 41) = 6.63$, $p = .014$, $\eta _p^2 = .14$, respectively.

## Declarations

### Open Practices

The data can be found in an online repository on osf: https://osf.io/mtrp9/?view_only=5ad6b97f287944debed9c3473e318e17. Correspondence should be addressed to Valentin Koob, koob@uni-bremen.de. None of the experiments was preregistered.
